# Human intestinal and circulating invariant natural killer T cells are cytotoxic against colorectal cancer cells via the perforin–granzyme pathway

**DOI:** 10.1002/1878-0261.13104

**Published:** 2021-10-21

**Authors:** Angélica Díaz‐Basabe, Claudia Burrello, Georgia Lattanzi, Fiorenzo Botti, Alberto Carrara, Elisa Cassinotti, Flavio Caprioli, Federica Facciotti

**Affiliations:** ^1^ Department of Experimental Oncology IEO European Institute of Oncology IRCCS Milan Italy; ^2^ Department of Oncology and Hemato‐oncology Università degli Studi di Milano Milan Italy; ^3^ Department of Pathophysiology and Transplantation Università degli Studi di Milano Milan Italy; ^4^ Department of Surgery Fondazione IRCCS Cà Granda Ospedale Maggiore Policlinico Milan Italy; ^5^ Gastroenterology and Endoscopy Unit Fondazione IRCCS Cà Granda Ospedale Maggiore Policlinico Milan Italy

**Keywords:** CD1d, colorectal cancer, cytotoxicity, iNKT, perforin

## Abstract

Invariant natural killer T (iNKT) cells are lipid‐specific T lymphocytes endowed with cytotoxic activities and are thus considered important in antitumor immunity. While several studies have demonstrated iNKT cell cytotoxicity against different tumors, very little is known about their cell‐killing activities in human colorectal cancer (CRC). Our aim was to assess whether human iNKT cells are cytotoxic against colon cancer cells and the mechanisms underlying this activity. For this purpose, we generated stable iNKT cell lines from peripheral blood and colon specimens and used NK‐92 and peripheral blood natural killer cells as cell‐mediated cytotoxicity controls. *In vitro* cytotoxicity was assessed using a panel of well‐characterized human CRC cell lines, and the cellular requirements for iNKT cell cytotoxic functions were evaluated. We demonstrated that both intestinal and circulating iNKT cells were cytotoxic against the entire panel of CRC lines, as well as against freshly isolated patient‐derived colonic epithelial cancer cells. Perforin and/or granzyme inhibition impaired iNKT cell cytotoxicity, whereas T‐cell receptor (TCR) signaling was a less stringent requirement for efficient killing. This study is the first evidence of tissue‐derived iNKT cell cytotoxic activity in humans, as it shows that iNKT cells depend on the perforin–granzyme pathway and both adaptive and innate signal recognition for proper elimination of colon cancer cells.

## Graphical abstract

Invariant natural killer T (iNKT) cells are lipid‐specific T lymphocytes with important roles in antitumor immunity. Here, we demonstrated that intestinal and blood‐derived iNKT cells can eliminate human colon cancer (CRC) cells. We also showed that iNKT cells require the release of perforin and granzymes to kill CRC cells, using both innate and adaptive mechanisms to recognize them.

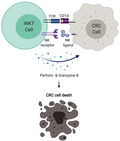

**Abbreviations**
CD1d: cluster of differentiation 1d
CRC: colorectal cancer
iNKT: invariant natural killer T cells
LPMC: lamina propria mononuclear cells
NK: natural killer cells
PBMC: peripheral blood mononuclear cells
TCR: T‐cell receptor

## 1 Introduction

Colorectal cancer (CRC) is the third‐most common cancer worldwide and the fourth cancer type for mortality [1]. Surgery, chemotherapy, and radiotherapy are currently the most frequently used therapeutic approaches for metastatic and nonmetastatic diseases. Targeted therapies with monoclonal antibodies directed against growth and angiogenic factors have been effectively included in the therapeutic options for CRC patients [1]. More recently, immunotherapy has gained consideration for CRC treatment thanks to the advent of technologies such as immune checkpoint inhibition [2, 3, 4] and CAR‐T technology [5, 6, 7, 8, 9]. Alongside CD8+ cytotoxic T lymphocytes [10, 11, 12] and natural killer (NK) cells [13, 14], innate‐like T lymphocytes, including invariant natural killer T (iNKT) cells [15], mucosa‐associated invariant T cells (MAIT) [16, 17], and gamma delta T cells [18, 19, 20], have been taken into consideration for cancer immunotherapy. iNKT cells show unique features classically associated with innate immunity, such as tumor‐directed cytotoxicity via innate receptors (i.e., NKG2D), but they also express a T‐cell receptor which might specifically recognize tumor‐derived antigens [16, 21, 22].

Invariant natural killer T cells are a nonconventional T‐cell population coexpressing natural killer lineage receptors and a semi‐invariantly rearranged, CD1d‐restricted, lipid‐specific T‐cell receptor [23]. In fact, iNKT cells can be activated in an adaptive manner by microbial and endogenous lipids presented by the CD1d antigen‐presenting molecule [24, 25, 26] and in an innate manner via activating NK receptors or cytokines, such as IL‐12 and IL‐18 [27, 28]. Thanks to their cytotoxic activity, iNKT cells are involved in antitumor functions against many types of cancers, showing either a direct or indirect antitumor effect on cancer cells [29, 30, 31, 32, 33, 34]. Murine iNKT cells isolated from liver, thymus, and spleen can kill leukemia, melanoma, lung, breast, and colorectal cancer cells both *in vitro* and *in vivo*; they express Fas ligand (FasL), TNF‐related apoptosis‐inducing ligand (TRAIL), granzyme B (GZMB), and perforin (PRF) and can target tumor cells in a CD1d‐dependent or CD1d‐independent manner [29, 33, 35, 36, 37, 38, 39, 40, 41]. While hematopoietic tumors express CD1d, many solid tumors are CD1d negative but can nonetheless be targeted by other mechanisms, including NKG2D engagement [27, 37, 42], or by indirect activation of iNKT cells by other CD1d‐expressing, antigen‐presenting cells present within the tumor microenvironment [31]. *In vitro* studies have shown that the preferred killing mechanism adopted by iNKT cells is the granzyme/perforin pathway [27, 36, 42, 43, 44]. However, the death receptor pathways (involving FasL and TRAIL) are also used *in vivo* [35, 38, 41].

A role for iNKT cells in killing human cancers has been demonstrated [34, 36, 37, 42, 45, 46], and Va24+ NKT cell tumor infiltration is considered an independent positive prognostic factor in human CRC [47]. Nonetheless, the data generated so far in human cancers describe only the cytotoxic activity of blood‐derived iNKT cells [21, 33, 36, 39, 40, 45, 48], possibly as a consequence of the great difficulty to isolate and perform functional assays with human tissue‐derived iNKT cells. As iNKT cells are mostly tissue‐resident and not recirculating [49], and the presence of different iNKT cell subsets (analogous to the T helper classification) seems to differ in the mouse [50], it is of great interest to study how tissue‐derived and circulating iNKT cell activities might differ. Moreover, very little is known about iNKT cell‐mediated killing mechanisms in human colorectal cancer, and if differences exist in terms of cytotoxic activities between peripheral blood and gut‐derived iNKT cells. In murine models of colon cancer, iNKT cells seem to have contrasting roles, as they promote polyp formation in Apc^Min−/+^ mice by producing IL‐10 [51] but decrease tumor progression in AOM/DSS‐treated mice by inducing a reduction of IL‐13 [52]. Given the differences between mice and humans, it is important to address how iNKT cells behave in the context of human colon cancer.

Here, we demonstrate that not only circulating but also intestinal iNKT cells are cytotoxic against human colon cancer cell lines as well as patient‐derived CRC cells. We showed that upon coculture with colorectal cancer cells, peripheral and intestinal iNKT cells expressed TRAIL, Fas ligand, and released granzyme B and perforin. Mechanistically, iNKT cell cytotoxicity against human CRC is preferentially perforin and granzyme‐dependent, but not completely CD1d‐mediated. These results provide the first insights about the cytotoxic role of human intestinal iNKT cells against CRC and contribute to shed light on the implementation of these cells for immunotherapeutic approaches against colorectal cancer.

## 2 Materials and methods

### 2.1 Human CRC cell lines

Caco‐2, HT‐29, RKO, DiFi, Colo 205 (from American Type Culture Collection, ATCC), NCM460D (from INCELL), and NK‐92 (from DSMZ) were cultured with specific media according to Table S1.

### 2.2 Human PBMC and LPMC isolation

Healthy donors’ peripheral blood buffy coats were obtained from San Matteo Hospital, Pavia, Italy, whereas intestinal specimens were obtained from IRCCS Policlinico Ospedale Maggiore, Milan, Italy. Institutional Review Board (Milan, Area B) approved the study with permission number 566_2015. All methodologies were under full compliance with the Declaration of Helsinki, and informed consent was obtained from all subjects.

Peripheral blood mononuclear cells (PBMC) were isolated by density gradient centrifugation using Ficoll‐Paque as for standard protocol. Natural killer cells were isolated from PBMCs using the NK Isolation Kit (Miltenyi Biotec, Bergisch Gladbach, Germany). Human lamina propria mononuclear cells (LPMCs) were isolated as previously described [53]. Briefly, the dissected intestinal mucosa was depleted of mucus and epithelial cells in sequential washes with Hanks’ Balanced Salt Solution (HBSS, Euroclone, Pero, Italy) containing DTT (0.1 mmol·L^−1^, Sigma‐Aldrich, St Louis, MO, USA) and EDTA (1 mmol·L^−1^, Sigma‐Aldrich) and then digested with collagenase D (400 U·mL^−1^, Worthington Biochemical Corporation, Lakewood, NJ, USA) for 5 h at 37 °C. LPMCs were then separated with a Percoll gradient and cultured in RPMI‐1640 medium containing 5% human serum (Sigma‐Aldrich), penicillin/streptomycin, gentamicin, and 100 U·mL^−1^ IL‐2 (Proleukin).

### 2.3 Isolation of CRC cells from surgical specimens

Colorectal cancer specimens were obtained from IRCCS Policlinico Ospedale Maggiore, Milan, Italy, under the same permission number as in 2.2. Specimens’ mucosa underwent sequential washes with Hanks’ Balanced Salt Solution (HBSS, Euroclone) containing DTT (0.1 mmol·L^−1^, Sigma‐Aldrich) and EDTA (1 mmol·L^−1^, Sigma‐Aldrich) in the presence of penicillin/streptomycin and gentamicin. Epithelial cells were obtained after collecting the supernatants from washes with EDTA, centrifuging at 200× ***g*** for 5 min, and preserving the pellet. Purity was confirmed via flow cytometry.

### 2.4 Human iNKT cell isolation and stable line generation

All the iNKT cell lines generated for this study are listed in Table S2. iNKT cells were isolated from LPMC and PBMC cells by sorting CD45^+^ CD3^+^ CD1d : PBS‐57Tet^+^ cells as described in Fig S1 [54]. For stable line generation, sorted iNKT cells were stimulated with phytohemagglutinin (PHA, 1 µg·mL^−1^, Sigma‐Aldrich) and irradiated peripheral blood feeders in a 2 : 1 iNKT : feeder ratio. PBMCs used as feeders were irradiated at 12.5 Gy and not pulsed with α‐GalCer. We did not stimulate iNKT cells in the presence of α‐GalCer to prevent iNKT cells to skew toward a Th1 phenotype as previously reported [55]. Stimulated cells were then expanded for 15 days by subculturing them every 2–3 days and maintained in RPMI‐1640 medium with stable glutamine, 5% v/v human serum, and 100 IU·mL^−1^ IL‐2 (Proleukin).

### 2.5 Assessment of cell viability

Cell viability prior and after coincubation was addressed using the Zombie Green Fixable Viability Kit (BioLegend, San Diego, CA, USA) according to the manufacturer’s instructions. In brief, cells were stained with Zombie Green dye and incubated for 20 min at room temperature, and subsequently washed twice with 1% BSA in PBS.

### 2.6 Lactate dehydrogenase release assay

Cytotoxicity was assessed using the Cytotoxicity Lactate Dehydrogenase (LDH) Assay Kit‐WST (nonhomogeneous assay, Dojindo, EU) following the manufacturer’s instructions. All experimental conditions were performed in duplicate. Cancer cells (2.5 × 10^4^ cells·well^−1^) were incubated at 37 °C for 4 h with effector cells at effector : target (E : T) ratios of 8 : 1, 4 : 1, 2 : 1, and 1 : 1. Supernatants were collected and plated in optically clear, 96‐well plates, and absorbance at 490 nm was measured using a GloMax Microplate Reader (Promega, Madison, WI, USA) after the colorimetric reaction for LDH detection was finished. The percentage of cytotoxicity was calculated as follows: (test well – spontaneous release control)/(maximal release control – spontaneous release control) × 100. For blocking experiments, iNKT cells were used at 8 : 1 E : T ratio. Target cells were pretreated for 30 min with anti‐CD1d antibody (10 µg·mL^−1^, clone CD1d42, R&D Systems); iNKT cells were pretreated with anti‐Fas Ligand antibody (30 min, 10 µg·mL^−1^, clone 100419, R&D Systems), anti‐TRAIL antibody (30 min, 10 µg·mL^−1^, clone 75411, R&D Systems), concanamycin A (2 h, 10 nm, Sigma‐Aldrich), or 3,4 dichloroisocoumarin (20 min, 40 µm, Enzo Life Sciences). Media from pretreated cells was then removed and replaced with fresh medium.

### 2.7 Calcein release assay

Target cell death was also addressed using the Calcein AM Assay Kit (Abcam, Cambridge, UK). All experimental conditions were performed in duplicate. In summary, 2.5 × 10^4^ cells·well^−1^ were incubated with Calcein AM for 30 min at 37 °C and then washed with PBS. Then, iNKT cells were added to each well at 8 : 1 E : T ratio for 4h. Supernatants were collected and transferred to 96‐well, white opaque plates (Falcon), and fluorescence was measured at Ex: 475 nm, Em: 500–550 nm using a GloMax Discover Microplate reader (Promega). The percentage of cytotoxicity was calculated as follows: (test well – spontaneous release control)/(maximal release control – spontaneous release control) × 100.

### 2.8 Caspase‐3/7 activity assay

Caspase‐3 and caspase‐7 activity was measured using the ApoLive‐Glo Multiplex Assay (Promega) according to the manufacturer’s instructions. All experimental conditions were performed in duplicate. iNKT cells were used in an 8 : 1 E : T ratio and were incubated with cancer cells (12.5 × 10^4^ cells·well^−1^) at 37 °C for 4 h in 96‐well, white opaque plates (Falcon). After coculture, luminescence was measured with a GloMax Microplate Reader (Promega). Ionomycin (Sigma‐Aldrich, 100 mm, 6 h) was used as necrosis control. Controls also included untreated cancer cells, effector cells alone, and wells with medium only. Luminescence upon coculture was calculated as follows: Test well – mean (effector cells alone wells).

### 2.9 Flow cytometry

Cells were stained with combinations of directly conjugated antibodies as specified in Table S3. The gating strategies to identify and analyze effector and target cells are described in Fig. S1. Effector cells were fixed and permeabilized with Cytofix/Cytoperm (BD) and treated with brefeldin A before the addition of antibodies, whereas colon cancer cells were fixed with 1% paraformaldehyde (PFA, Sigma‐Aldrich). All samples were acquired with a FACSCelesta Cytometer using the BD facsdiva software (BD Biosciences Milan, Italy). Analyses were performed using the flowjo software version 10.5.3 (BD Biosciences).

### 2.10 Cytotoxicity molecule release measurements

Granzyme B (GZMB, MabTech, Cambridge, UK) and perforin (PRF, MabTech) concentrations in coculture supernatants were measured by sandwich ELISA assays according to manufacturers’ protocols (for antibodies, Table S4). All experimental conditions were performed in duplicate. Absorbance was measured at 450 nm using a GloMax Microplate Reader (Promega).

### 2.11 Statistical analysis

Analysis of variance (ANOVA) and Tukey and Šidak’s tests for multiple comparisons or Kruskal–Wallis test with Dunn’s test for multiple comparisons and graphs were done with graphpad Prism version 8.0. Linear regressions and cytotoxicity percentages were calculated with Microsoft Excel version 16.25. *P* < 0.05 (*), 0.01 (**), 0.001 (***), and 0.0001 (****) were regarded as statistically significant.

## 3 Results

### 3.1 Human Intestinal and circulating iNKT cells are cytotoxic against colon cancer cell lines

**Fig. 1:**
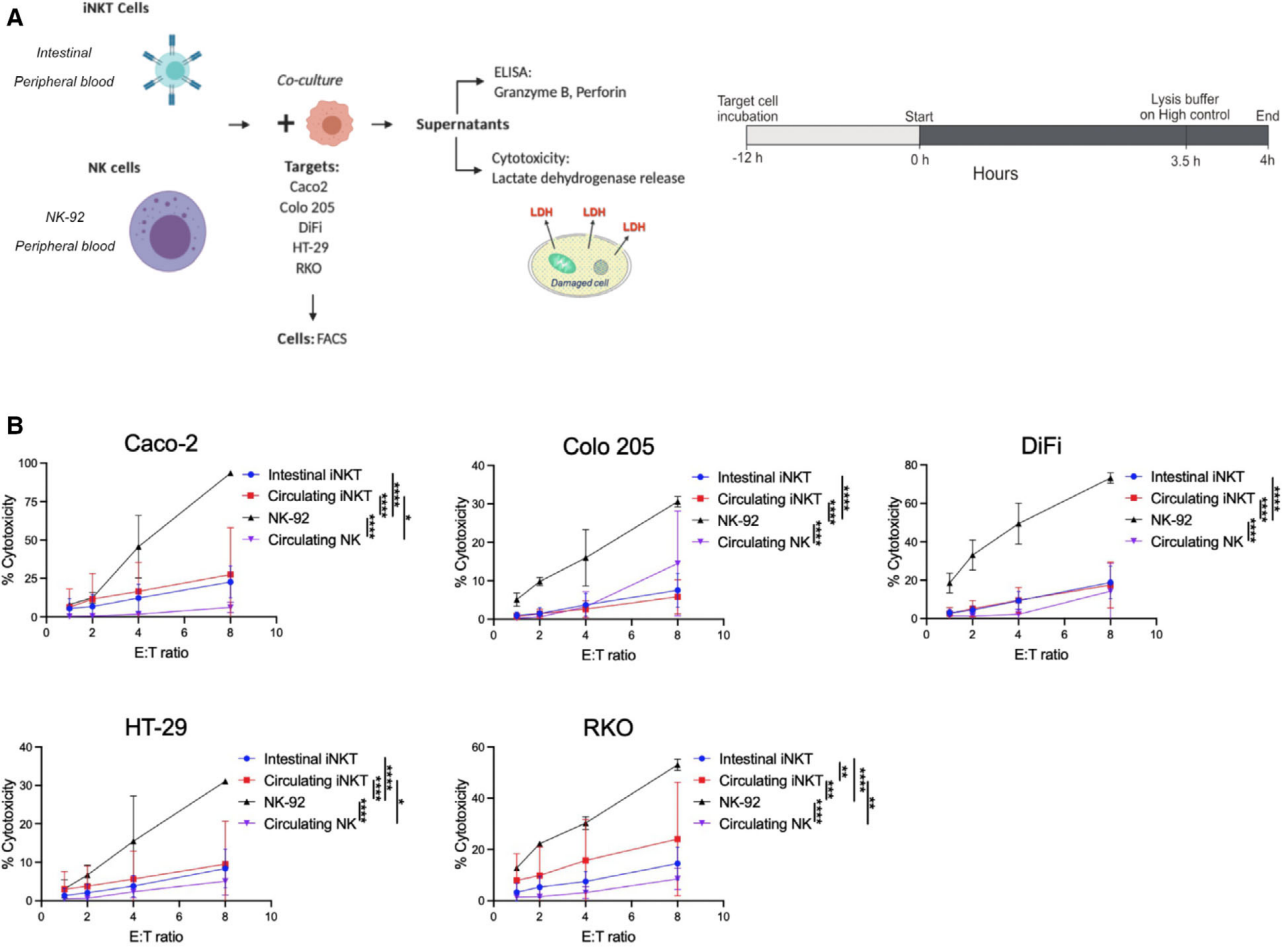
iNKT cell lines exert direct cytotoxic activities against colon cancer cell lines. (A) Experimental design. (B) Effector : target (E : T) ratio curve graphs comparing the killing efficiency of intestinal and circulating iNKT cells (pooled donors) with NK‐92 and peripheral blood natural killer (NK) cells. Two‐way ANOVA tests were used to assess statistical significance and Tukey tests for multiple comparisons. Data are means ± SD of at least 3 independent experiments for iNKT and NK‐92 cell experiments. ns. nonsignificant, *P*‐value < 0.05 (*), 0.01 (**), 0.001 (***), 0.0001 (****).

**Fig. 2:**
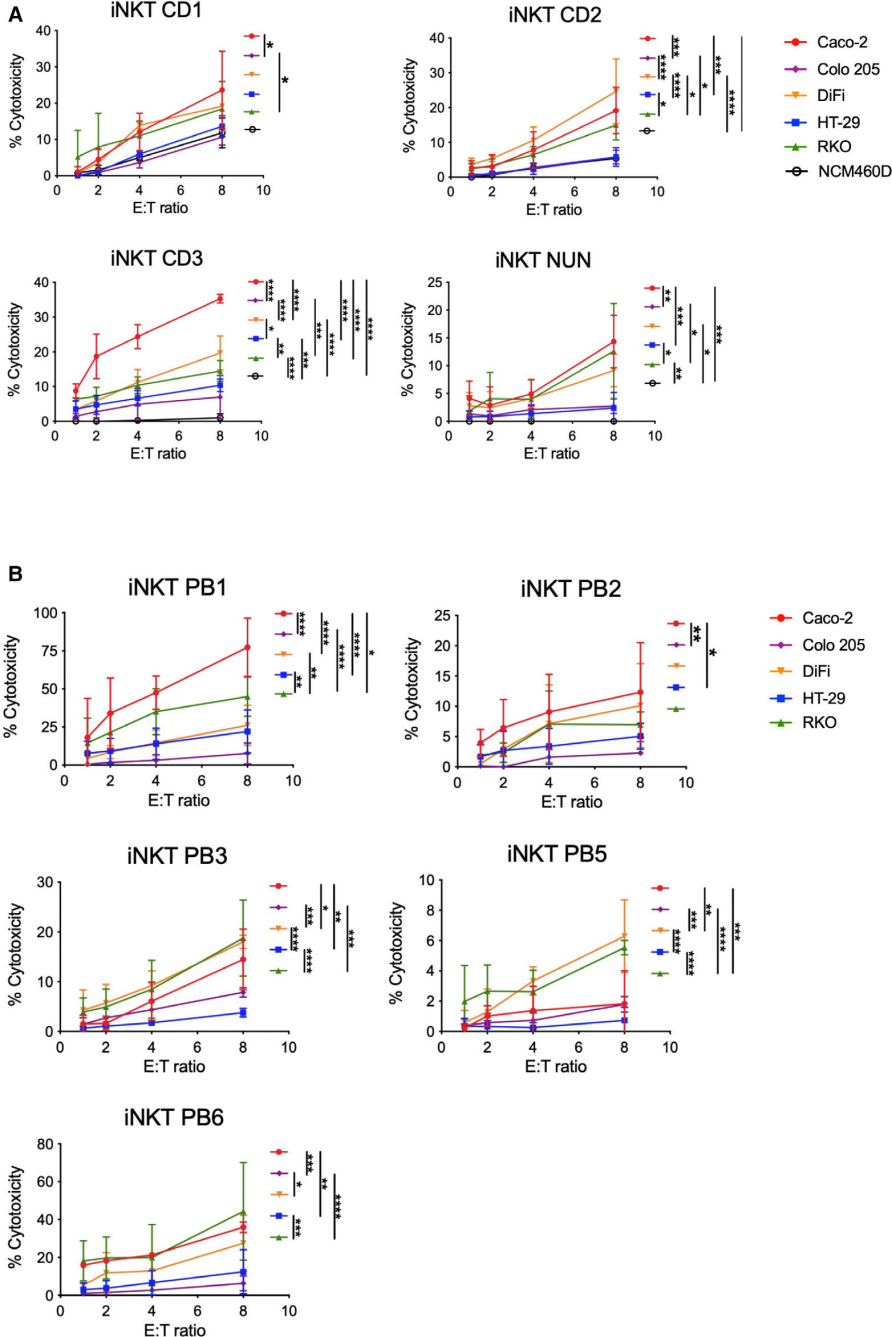
iNKT cells kill colon cancer cell lines with different efficiencies. (A–B) E : T ratio curves of each intestinal (A) and circulating (B) iNKT cell line comparing killing levels for each target cell line. Two‐way ANOVA tests were used to assess statistical significance and Tukey tests for multiple comparisons. Data are means ± SD of at least 3 independent experiments. ns, nonsignificant, *P*‐value < 0.05 (*), 0.01 (**), 0.001 (***), 0.0001(****).

Human peripheral blood iNKT cells exert cytotoxic functions against cancers of different origins [21, 33, 34, 39, 45, 46]. To test whether iNKT cells of either blood or intestinal origin are capable of cytotoxic against colon cancer cells, we isolated iNKT cells by cell sorting, expanded them *in vitro,* and generated stable lines to perform functional assays (Fig. S1). Several stable cell lines from healthy peripheral blood and intestinal specimens were obtained (*n* = 9, Table S2). Circulating (PB1, PB2, PB3, PB5, and PB6) and intestinal (CD1, CD2, CD3, and NUN) iNKT cells were coincubated with five colorectal cancer cell lines at different effector : target ratios for *in vitro* cytotoxicity evaluation by measuring lactate dehydrogenase release (Fig 1A). As this assay measures the release of the whole system, we also measured iNKT cell viability prior and after coincubation using Zombie staining (Fig. S2E). For these series of experiments, NK‐92 cells, a stable human natural killer cell line, and freshly isolated, circulating NK cells were used as cell‐mediated cytotoxicity controls (Figs. 1A, Fig. S2C & Fig. S3A). Both intestinal (Fig. 2A) and circulating (Fig. 2B) iNKT cells exerted efficient killing activity against all colon cancer cell lines tested. Effector : target ratio curves for iNKT cell killing were similar to that of blood natural killer cells, but killing was lower compared with NK‐92 cells (Fig. 1A). When iNKT cell lines were analyzed separately, we observed great donor variability in killing efficiency, being iNKT CD1‐3, PB1, and PB6 the most efficient (Fig. S2A–B).

Albeit all cancer cell lines were targeted and killed by iNKT cells of different origins, cytotoxicity was not equal against the target lines. In general, Caco‐2, DiFi, and RKO cells were the most efficiently eliminated, whereas Colo 205 and HT‐29 cells were more resistant to iNKT cell killing (Fig. 2). This was also confirmed by low caspase activity and calcein release upon coculture with iNKT cells (Fig. S2D & F). The same pattern was observed on NK‐92 cells, but not on blood‐derived NK cells, as each donor displayed different killing preferences (Fig. S3A). As intestinal iNKT cells were obtained from patients with inflammatory bowel diseases (Table S2), we wondered whether these cells were cytotoxic due to a skewed proinflammatory phenotype and not being able to distinguish between normal and tumor colon cells. To test this, we performed killing experiments using a normal colon cell line (NCM460D) as target cells. We observed that none of the intestinal iNKT cells could eliminate NCM460D cells with the exception of iNKT CD1, which showed a mild killing activity (Fig. 2A).

Altogether, these data indicate that the tissue of origin does not define the cytotoxic potential of iNKT cell lines against colon cancer cells. Furthermore, we showed that some target lines are more prone to iNKT cell killing, suggesting differences in how tumor cells can activate iNKT cell cytotoxic activity.

### 3.2 iNKT cells change the expression of TRAIL and Fas ligand upon interaction of CRC cells

**Fig. 3:**
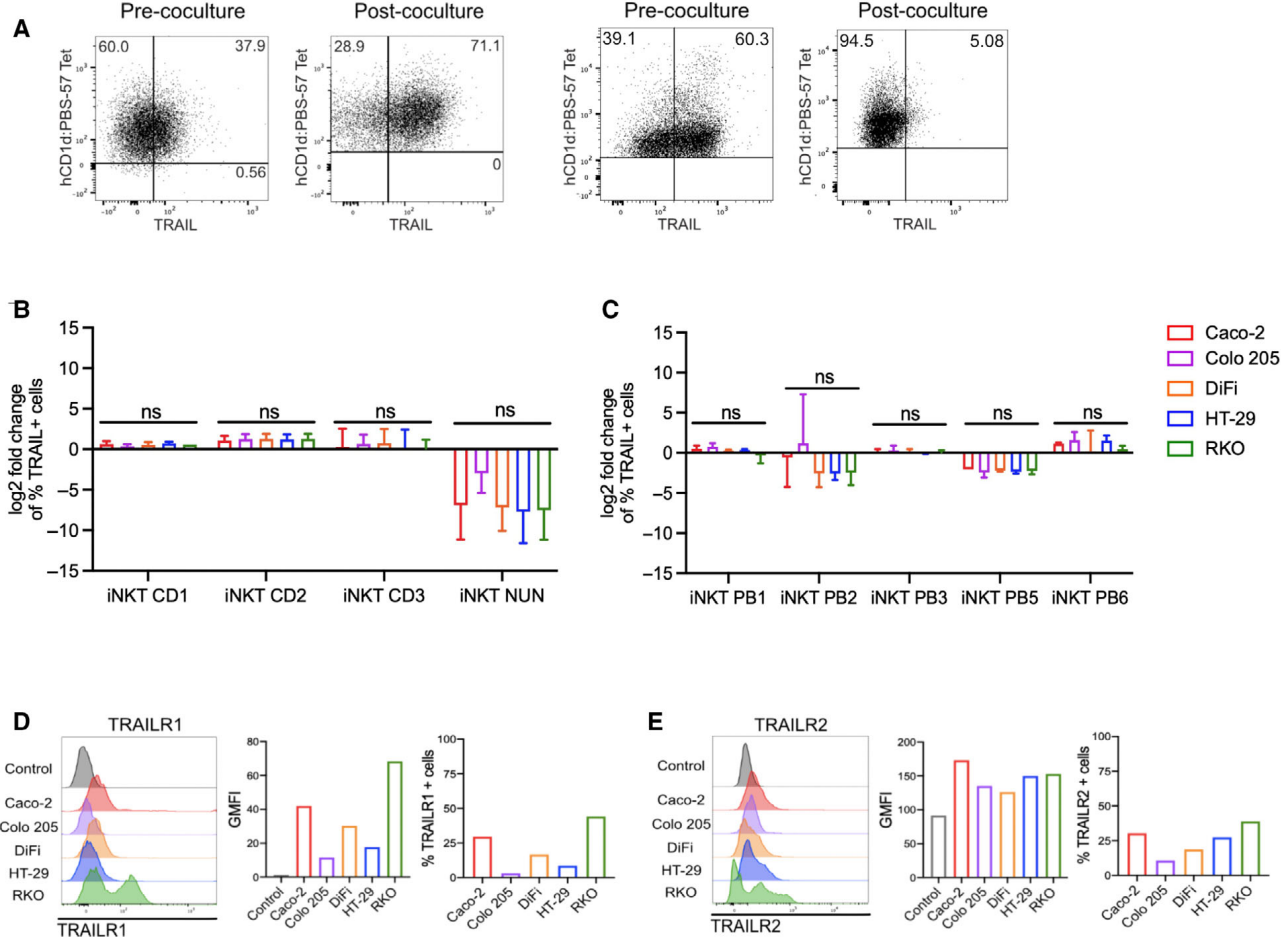
Surface TRAIL changes upon encounter with colon cancer cells. (A) Representative dot plots for iNKT cells prior and after coculture. (B–C) Fold change of TRAIL‐positive intestinal (B) and blood‐derived (C) iNKT cells respect to resting conditions. D‐E. TRAIL receptor 1 (TRAILR1, D) and TRAIL receptor 2 (TRAILR2, E) expression in colorectal cancer (CRC) cell lines. Kruskal–Wallis test was used to assess statistical significance, and Dunn’s test was used for multiple comparisons in B‐C. ns. nonsignificant, *P*‐value < 0.05 (*), 0.01 (**), 0.001 (***), 0.0001 (****). Data are means ± SD of at least 3 independent experiments in B and C.

**Fig. 4:**
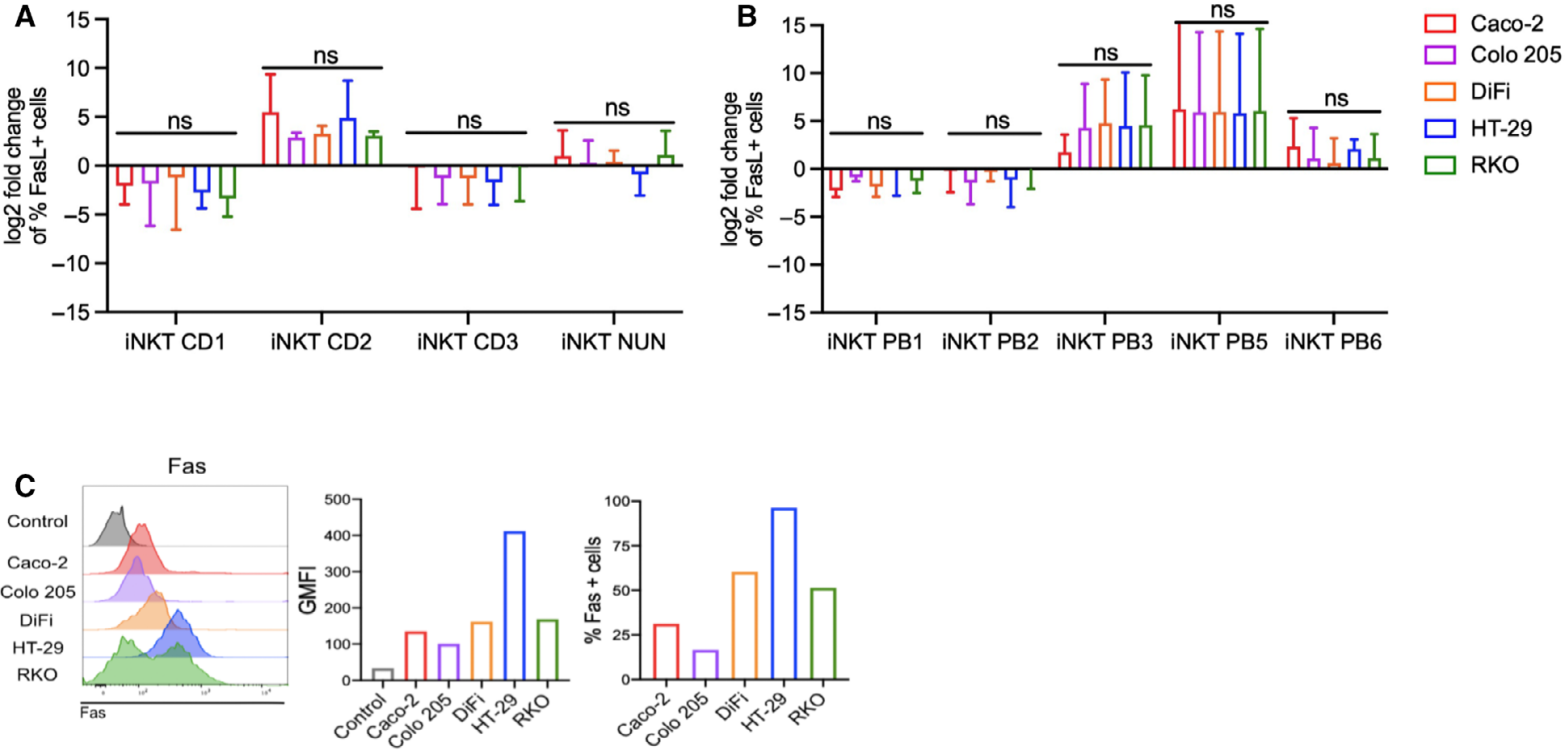
Fas ligand is modulated upon encounter with colon cancer cells. (A–B) Fold change of Fas ligand expression by colon (A) and circulating (B) iNKT cell lines at steady‐state conditions vs after 4‐h incubation with CRC cell lines. (C) Fas expression by colon cancer cells. Kruskal–Wallis test was used to assess statistical significance, and Dunn’s test was used for multiple comparisons. ns. nonsignificant, *P*‐value < 0.05 (*), 0.01(**), 0.001 (***), 0.0001 (****). Data are means ± SD of at least 3 independent experiments for A and B.

Murine and human iNKT cells display on their surface the cytotoxicity‐related molecules Fas ligand and TRAIL, used for their cancer‐killing function *in vitro* and *in vivo* [35, 38, 41, 44]. Therefore, we analyzed the expression of these molecules on human iNKT cells before and after the exposure to CRC cells (Figs. 3 & 4). TRAIL levels at steady‐state conditions varied greatly among donors, ranging from 60% to less than 1% in average (Fig. S2I). Fas ligand expression, on the contrary, was considerably low in all the iNKT cells studied (Fig. S2J).

Upon encounter with CRC cells, TRAIL expression increased on some iNKT lines, namely CD1, CD2, and PB6, whereas it decreased on iNKT NUN, PB2, and PB5 cells (Fig. 3B & C). FasL expression was also modulated upon coculture, increasing on iNKT CD2, PB3, PB5, and modestly on iNKT PB6 cells, and decreasing on iNKT CD1, CD3, PB1, and PB2 cells (Fig. 4A & B). To note, also NK cells were characterized by a low expression of Fas ligand at steady‐state conditions, which was further reduced upon encounter with CRC cells (Fig. S3C). TRAIL expression varied among NK cells, being the highest on NK‐92 cells (Fig. S3B). Upon coculture, TRAIL was slightly upregulated on NK PB1 cells, while it was downregulated on NK PB2 cells (Fig. S3C).

The expression of Fas and TRAIL receptors 1 and 2 (TRAILR1 and TRAILR2) was variable on target cells (Figs. 3D–E & 4C). Differences among lines were observed in TRAILR1 (Caco‐2 29.5%, Colo 205 2.98%, DiFi 16.68%, HT‐29 8.61%, RKO 44.2%; Fig. 3D) and TRAILR2 (Caco‐2 30.3%, Colo 205 10.65%, DiFi 18.7%, HT‐29 27.37%, RKO 38.83%; Fig. 3E). Caco‐2 and Colo 205 cells showed relatively low Fas expression (Caco‐2 31.2%, Colo 205 16.58%), and DiFi and RKO cells had intermediate frequencies (DiFi 60.38%, RKO 51.39%) while most HT‐29 were Fas positive (96.25%; Fig. 4C). Taken together, these data confirm that human iNKT cells modulate TRAIL and Fas ligand membrane expression upon encounter with CRC cells.

### 3.3 Targeting of CRC cells induces secretion of perforin and granzyme B by iNKT cells

**Fig. 5:**
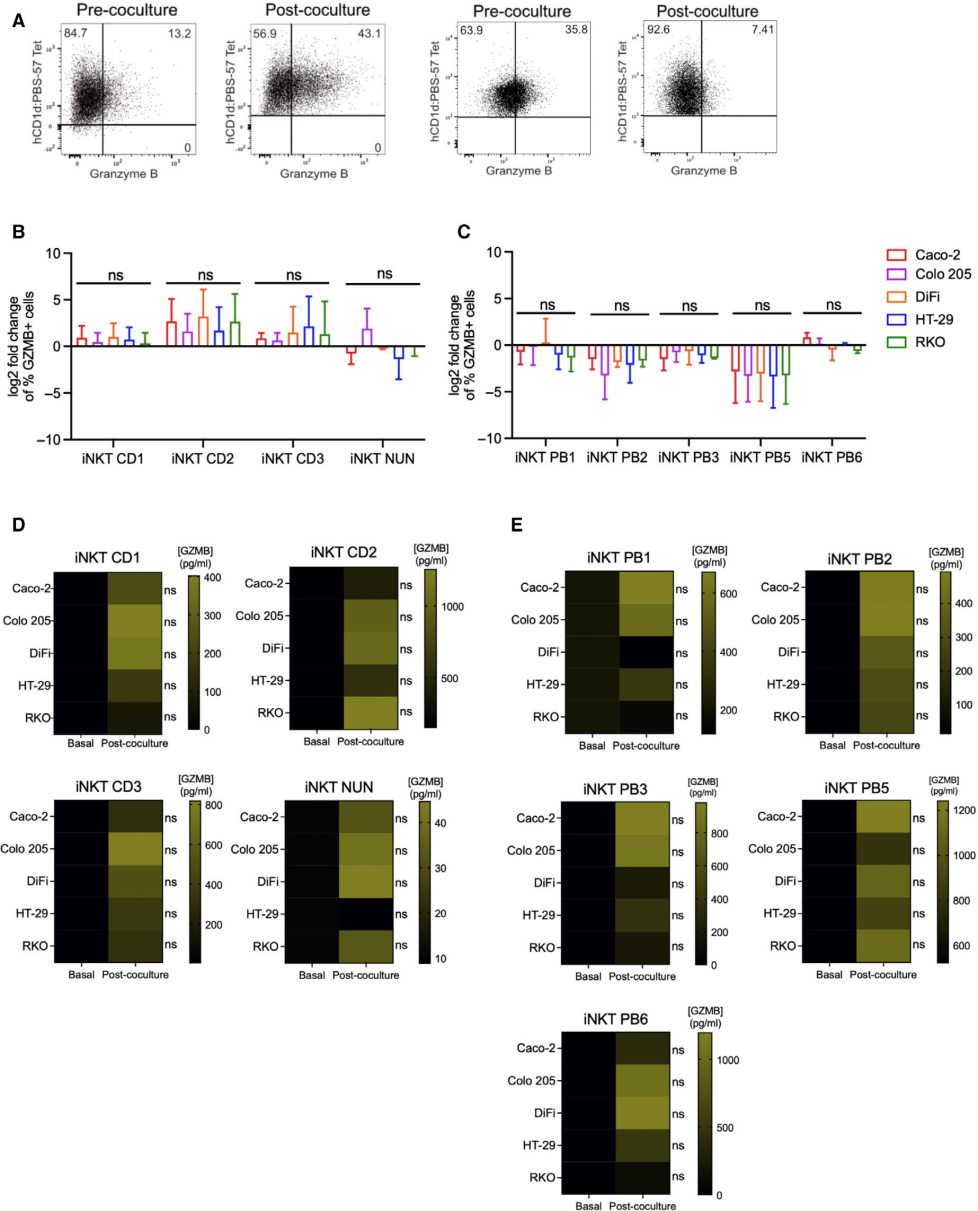
iNKT cell lines release granzyme B upon with CRC cell recognition. (A) Representative dot plots for iNKT cells prior and after coculture. (B–C) Fold change of frequencies of granzyme B (GZMB)‐positive intestinal (B) and circulating (C) iNKT cells respect to steady‐state conditions. D‐E. Granzyme B concentration in iNKT cell supernatants at 8 : 1 E : T ratio alone vs after incubation with colon cancer cells for intestinal (D) and peripheral blood (E) iNKT cells. Kruskal–Wallis test was used to assess statistical significance, and Dunn’s test was used for multiple comparisons. ns. nonsignificant, *P*‐value < 0.05 (*), 0.01 (**), 0.001 (***), 0.0001 (****). Data are means ± SD for (B–C) and means for (D–E) of 3 independent experiments.

**Fig. 6:**
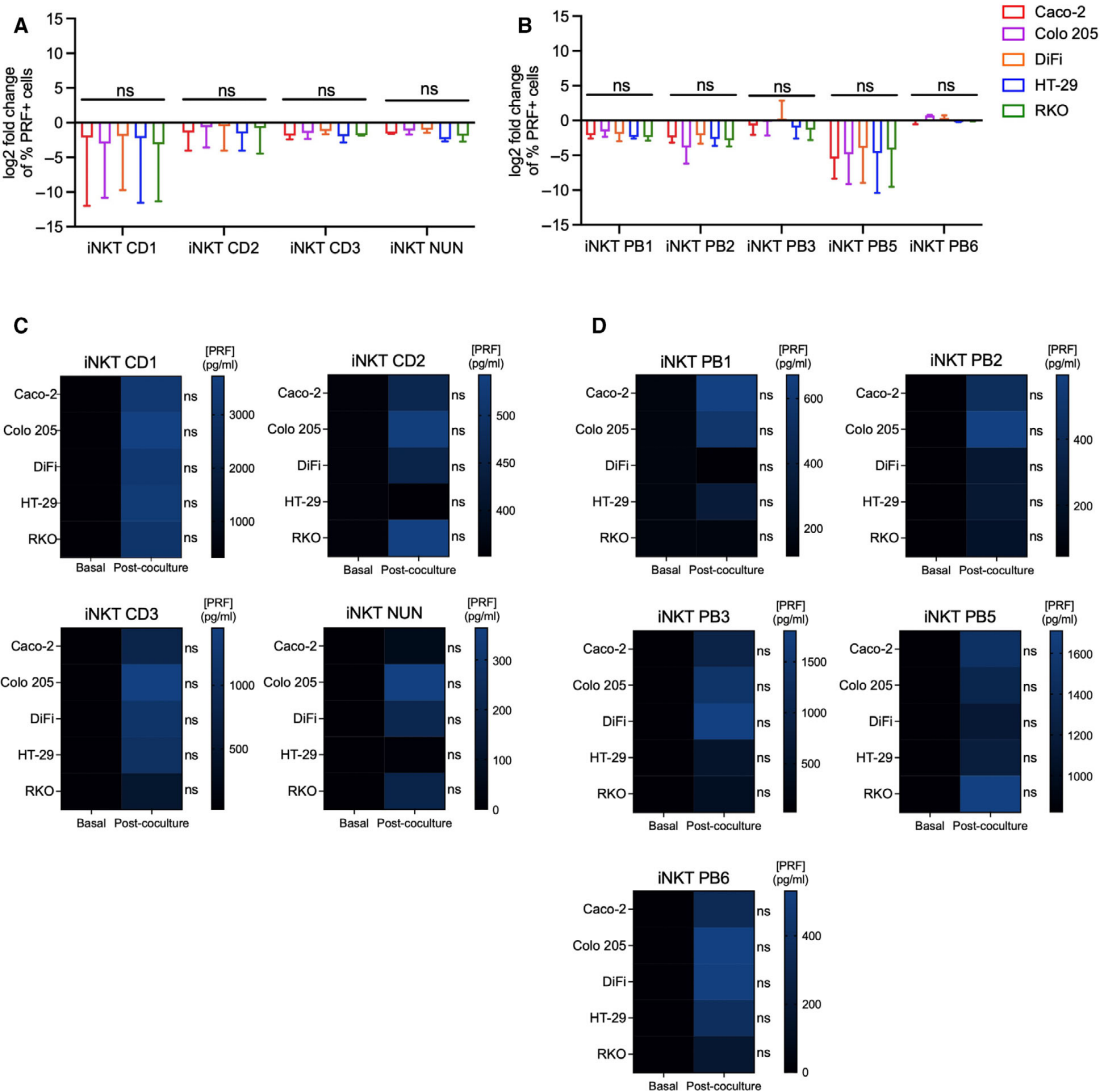
iNKT cell lines release perforin upon CRC cell encounter. (A–B) Frequencies of perforin (PRF)‐positive intestinal (A) and circulating (B) iNKT cells before and after 4‐h coincubation. (C–D) Perforin concentration in iNKT cell supernatants at 8 : 1 E : T ratio alone vs after incubation with colon cancer cells for intestinal (C) and peripheral blood (D) iNKT cells. Kruskal–Wallis test was used to assess statistical significance, and Dunn’s test was used for multiple comparisons. ns. nonsignificant, *P*‐value < 0.05 (*), 0.01 (**), 0.001 (***), 0.0001 (****). Data are means ± SD for B–C and means for D–E of 3 independent experiments.

Next, we assessed the production of soluble cytotoxic molecules prior and after coincubation of iNKT cells with colon cancer cells. Granzyme B (GZMB) and perforin (PRF) were detected by flow cytometry analysis and in culture supernatants. As it occurred with TRAIL and Fas ligand, frequencies of GZMB+ cells changed after incubation with colon cancer cells. More specifically, frequencies increased in iNKT CD1‐3 cells, whereas there was a reduction in almost all circulating iNKT cells with the exception of PB6, on which changes were quite modest (Fig. 5B–C). Perforin‐positive cells did not vary greatly upon coculture, decreasing in almost all the iNKT cell lines we tested (Fig. 5A & B). More importantly, to evaluate whether these molecules were also efficiently secreted, we measured granzyme B and perforin concentrations in coculture supernatants by ELISA and compared them with levels secreted by iNKT cells alone during the time of the killing experiments (basal level). Even if some iNKT cell lines could release spontaneously perforin and granzyme B, their levels were lower when compared to the release in the presence of CRC cells, being particularly evident in intestinal iNKT cells (Figs. 5D–E & 6C–D).

On the other hand, natural killer cells showed high frequencies of GRZB and PRF+ positive cells in steady state in some cases (Fig. S3D & F). Upon coculture, granzyme B‐positive cells mildly increased on NK‐92 and NK PB2 cells (Fig. S3D), whereas PRF‐positive cell frequencies increased in NK‐92 cells, but there was a reduction in NK PB2 cells (Fig. S3F). Interestingly, fresh NK cells secreted similar amounts of granzyme B as compared to iNKT cells, while perforin levels were higher (Fig. S3E & G). These results indicate that intestinal and circulating iNKT cells increase their secretion of granzyme B and perforin when they interact with CRC cells.

### 3.4 iNKT cells recognize CRC cells in CD1d‐dependent and CD1d‐independent manners, requiring perforin and granzymes for proper elimination

Murine and human iNKT cells can target and kill solid tumors by either antigen‐dependent or antigen‐independent mechanisms [21, 27, 33, 37, 38, 39, 42, 48]. We thus wondered which could be the case for human iNKT cells in the context of colorectal cancer. To test this, we used five of the most efficient iNKT cell lines and the three most sensitive CRC cell lines as target cells at the highest E : T ratio (8 : 1).

**Fig. 7:**
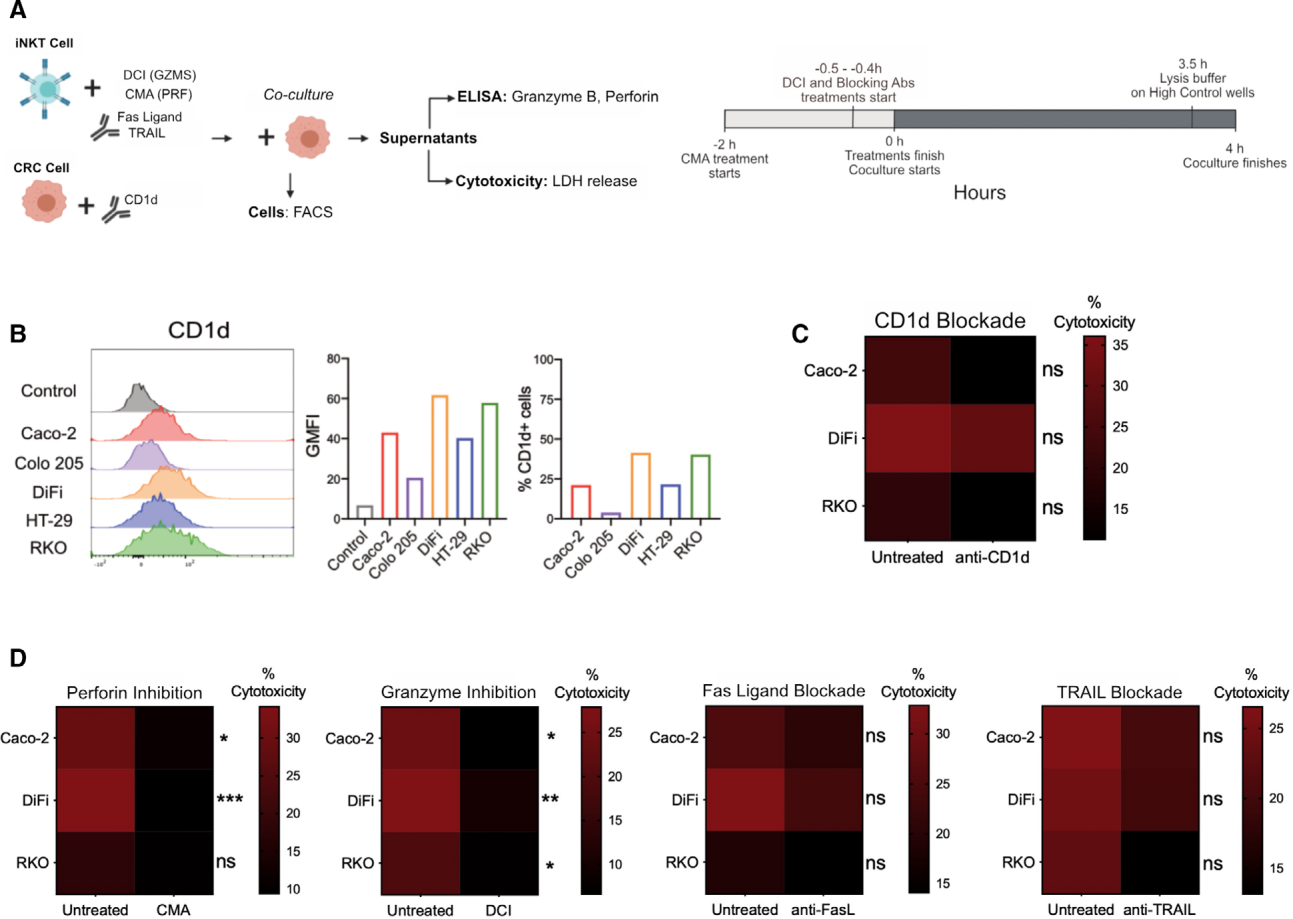
iNKT cells recognize CRC cells in CD1d‐dependent and CD1d‐independent manners, requiring perforin and granzymes for proper elimination. (A) Experimental design. All experiments were performed at an E : T ratio of 8 : 1. (B) CD1d expression in CRC cell lines. (C) Effect of CD1d blockade on iNKT cell elimination of CRC cells. (D) Effect of perforin, granzymes, Fas ligand, and TRAIL inhibition iNKT cell cytotoxicity. Two‐way ANOVA test was used to assess statistical significance, and Šidak’s test for multiple comparisons. ns. nonsignificant, *P*‐value < 0.05(*), 0.01 (**), 0.001 (*** 0.0001 (****). Data are means of five pooled iNKT cell lines with 3 independent experiments.

We first evaluated whether colon cancer cells expressed the CD1d molecule on their surfaces. All cell lines were CD1d positive, albeit differences in frequencies were found, with DiFi and RKO cells being the lines with most CD1d‐positive cells (DiFi 41.4%, RKO 40.3%) and the metastatic line Colo 205 having the lowest number of positive cells (3.75%; Fig. 7B). CD1d expression levels on these cells were lower than on professional antigen‐presenting cells such as monocyte‐derived dendritic cells and THP‐1 cells, a monocytic cell line (Fig. S1E–F). Interestingly, iNKT cell cytotoxicity was not profoundly affected by CD1d blockade that only induced a partial decrease in cytotoxic activities (Fig. 7C). These results suggest that iNKT cells can recognize CRC cells by both CD1d‐dependent and CD1d‐independent mechanisms.

Since antigen‐dependent mechanisms seemed to be only partially involved, to unravel the mechanism of iNKT cell cytotoxicity, we used inhibitors and blocking antibodies against different cytotoxicity‐related molecules prior to the coculture with cancer cells. Concanamycin A (CMA) was added to inhibit perforin, 3,4 dichloroisocoumarin (DCI) to inhibit granzymes, and neutralizing antibodies were used to block TRAIL (anti‐TRAIL) and Fas ligand (anti‐FasL) (Fig. 7D), for which we also assessed potential toxicity on iNKT cells by Zombie staining. At the concentrations used here, none of the inhibitors was toxic against iNKT cells (Fig. S4E). Perforin and granzyme inhibition drastically affected the cytotoxic functions of all the iNKT cell lines tested. Conversely, neutralizing antibodies induced a mild reduction in killing activity, being of particular interest the decrease induced by anti‐TRAIL antibody against RKO cells (Fig. 7D). To note, Fas Ligand, TRAIL, and CD1d blockade partially affected the release of granzyme B and perforin (Fig. S4A–B), while DCI treatment increased the frequencies of Fas ligand‐positive cells (Fig. S4C). Taken together, these results indicate that the perforin/granzyme pathway is required for iNKT cell cytotoxic activity against colon cancer cells.

### 3.5 iNKT cells kill patient‐derived colon cancer cells with production of perforin and granzyme B

**Fig. 8:**
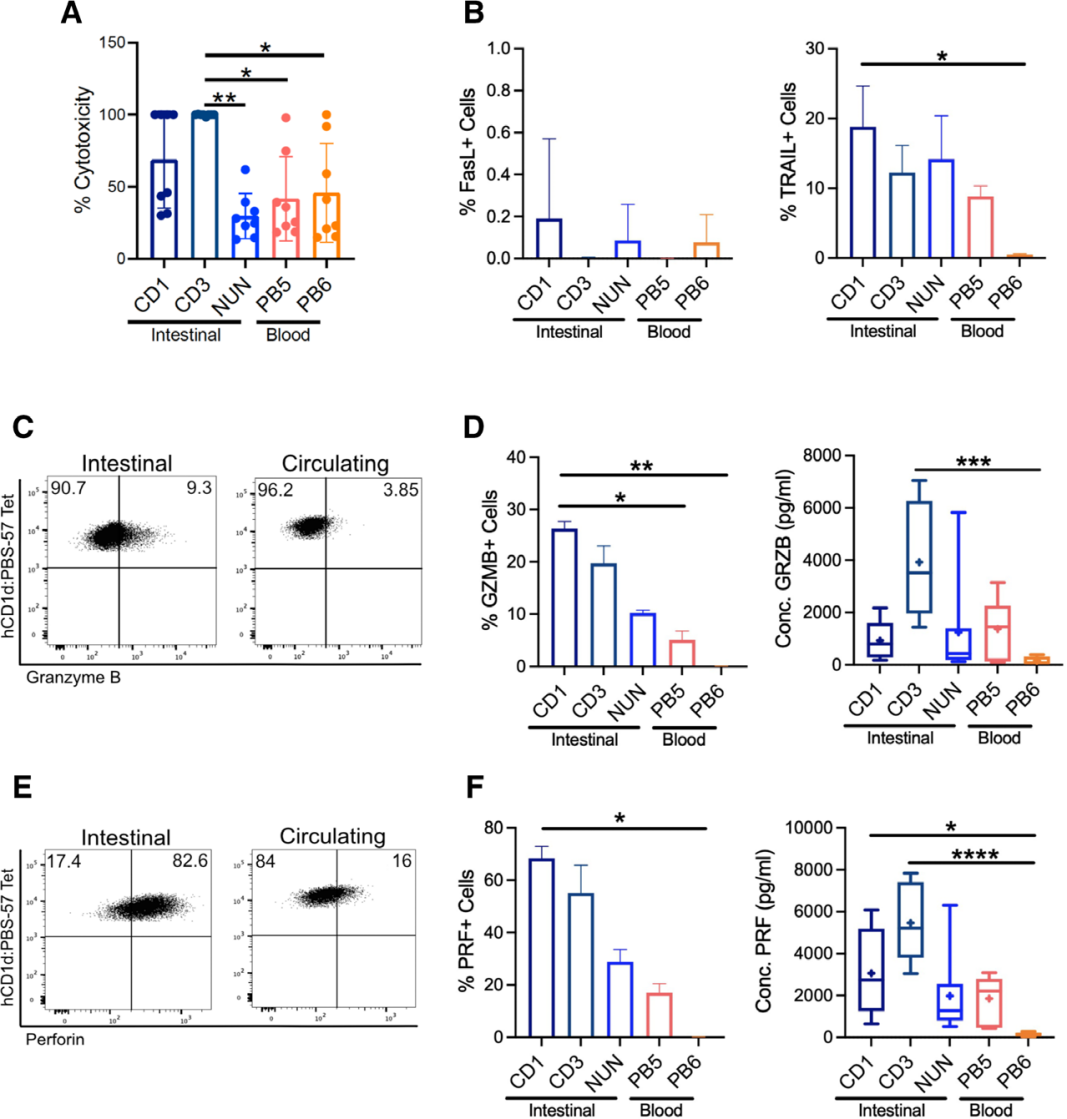
iNKT cells efficiently kill patient‐derived colon cancer cells. (A) Cytotoxicity at 8 : 1 E : T ratio. (B) Frequencies of Fas ligand (left) and TRAIL (right)‐positive iNKT cells after incubation with CRC cells (C) Representative dot plots showing granzyme B expression. (D) Frequencies of granzyme B‐positive cells (left) and granzyme B levels (right) after CRC cell encounter. (E) Representative dot plots showing perforin expression. (F) Frequencies of PRF‐positive cells (left) and perforin levels (right) after CRC cell encounter. Kruskal–Wallis test was used to assess statistical significance, and Dunn’s test was used for multiple comparisons. ns. nonsignificant, *P*‐value < 0.05 (*), 0.01 (**), 0.001 (***), 0.0001 (****). Data are means ± SD of 4 pooled patients.

As the previous experiments were performed with well‐established CRC cell lines, we wondered whether intestinal and peripheral blood iNKT cells were also efficient at eliminating cancer cells freshly isolated from CRC patients. To address this point, we isolated cancer cells from surgical specimens of four patients (Fig. S5A) and cocultured them with three intestinal and two circulating iNKT cell lines at 8 : 1 E : T ratio for 4 h. As shown in Fig. 8A, iNKT NUN and PB6 efficiently eliminated patient CRC cells, at comparable levels shown for CRC cell lines. More interestingly, iNKT CD1, CD3, and PB5 were more efficient at killing freshly isolated CRC cells than any of the cell lines previously tested. Strikingly, iNKT CD1 and CD3 reached 100% of killing efficiency for some patients (Figs. 8A & 2A–B). To note, all iNKT cell lines were characterized by the expression and release of granzyme B and perforin (Fig. 8D & F), whereas some of them upregulated death ligands (Fig. 8B). These data confirm that human intestinal and circulating iNKT cells have the ability to eliminate freshly isolated, patient‐derived colon cancer cells by releasing granzyme B and perforin.

## 4 Discussion

The antitumor functions of iNKT cells have been of particular interest since the emergence of cancer immunotherapies. They are peculiar in several ways, since they are capable of being activated by lipid antigens, innate signals, and cytokines, and not being affected by histocompatibility issues, as CD1d is a nonpolymorphic molecule [21, 37, 56, 57]. Nonetheless, the mechanisms of human iNKT cell cytotoxicity are still largely unknown. Here, we showed that human intestinal and peripheral blood‐derived iNKT cells exerted killing activities against colon cancer cells through different mechanisms, including granzyme B and perforin release and death receptor pathways.

Previous studies showed that human peripheral blood iNKT cells could have killing activities against cell lines from different origins, including colorectal cancer [34, 39, 42, 44, 45, 58]. Here, we observed that both circulating and intestinal iNKT cells could kill all the colon cancer cell lines they were exposed to. This study is the first that demonstrates the cytotoxic activity of human intestinal iNKT cells. Furthermore, we showed that the perforin/granzyme pathway is required for iNKT cell cytotoxic functions against colorectal cancer cells.

iNKT cells are usually divided into subsets depending on their transcriptional programs and cytokines released [49]. The NKT1 subset is of particular importance in cancer research as it is the only iNKT cell subset endowed of antitumor activities, both through cytokine release and cytotoxic activity [49]. Studies in mice have shown that these cells are mainly located in the liver, intestine, thymus, lung, and spleen [49, 59],suggesting that iNKT cells from many organs are potentially capable of killing transformed cells [38, 40, 41, 44, 46]. In addition, most of the human iNKT cell cytotoxicity studies have been performed with cells derived from peripheral blood [21, 34, 36, 39, 45, 46, 48], indicating that iNKT cells from different tissues preserve their cytotoxic activity, being capable of eliminating tumor cells even if these do not belong to the same tissue/organ of origin. In line with these findings, we observed that peripheral blood and intestinal iNKT cell lines were similarly efficient at killing colorectal cancer cells. It is noteworthy that intestinal iNKT cell lines were obtained from tissue samples from inflammatory bowel disease (IBD) patients, known to be characterized by the intestinal infiltration of pathogenic Th1/17 lymphocytes [60], including iNKT cells [54]. However, even if our intestinal iNKT lines have the potential of being proinflammatory and tissue‐damaging, we observed that they were not cytotoxic against normal colon cells, discarding the possibility that the observed cytotoxicity was due to their proinflammatory origin.

From a mechanistic point of view, iNKT cells, similarly to other cytotoxic populations, are known for inducing tumor cell death both by granzyme B/perforin production and by expression of Fas ligand (FasL) and TRAIL on their plasma membranes [38, 42, 44, 61, 62, 63]. Here, all the iNKT cell lines, regardless their origin, produced granzyme B and perforin to eliminate CRC cell lines. In particular, we confirmed that the prevailing killing mechanism of iNKT cells was based on the presence of functional perforin and granzymes, as treatment with concanamycin A and DCI significantly impaired iNKT cell killing against the colon cancer lines tested. While the perforin/granzyme pathway does not require the presence of specific receptors on target cells, the death ligand pathways require death receptor expression and are slower than the granzyme/perforin mechanism, requiring more degranulation events [61, 64]. This might explain why the PRF/GZMB mechanism was prevalent in this model. Even if the presence of functional perforin and granzyme B is fundamental for iNKT cell killing of CRC cells, some iNKT lines expressed TRAIL on their membranes, and some of them upregulated Fas ligand upon encounter with colon cancer cells. Furthermore, Fas and TRAIL receptors were present in CRC cell lines. Thus, it is possible to envisage that upon cancer cell encounter, cytotoxic cells might choose the best killing mechanism according to their activation state and/or by switches from PRF/GZMB to death receptor pathways over time, as it occurs with natural killer cells [63].

Although iNKT cell cytotoxicity was not profoundly affected by CD1d blockade, killing was still reduced. These results indicate that epithelial cancer cells not only express CD1d on their membranes, but they might also present antigens involved in the adaptive activation of iNKT cell cytotoxicity, even if not as strongly as other innate signals. In this regard, iNKT cells are known to be activated by a range of self‐antigens [56, 65, 66, 67, 68]. Altered pathways observed in cancer might affect lipid presentation and therefore iNKT cell activation. For example, it has been observed that endoplasmic reticulum stress, process commonly found in cancer cells, induces the enhancement of immunogenic self‐lipid antigens and activation of iNKT cells [56, 68]. While CD1d presence is essential for iNKT cell killing in some cancer types [38, 40, 42, 44, 45] other studies, including this one, have demonstrated that in some contexts, antigen presentation by cancer cells might not be as important as triggering by innate mechanisms [27, 36, 37, 46, 69].

Here, we also observed that CRC lines were not eliminated with the same efficiency, being some lines more sensitive than others to iNKT cell‐mediated killing. These patterns of sensitivity were considerably preserved among iNKT cell lines and NK‐92 cells, suggesting similar mechanisms of detection of tumor cells by iNKT cell lines from different tissue origins. Moreover, iNKT cell cytotoxicity was not substantially affected by the inhibition of CD1d. Both phenomena can be explained by the innate nature of iNKT cells, and the characteristics displayed by different CRC cells. One of the main features of iNKT cells is the expression of some NK receptors. On natural killer cells, the balance given by the interactions of activating and inhibitory receptors with their respective ligands establishes their activation state [70]. iNKT cells might be also regulated by the same mechanisms, as they express NKG2D, NKG2A, NKp30, NKp46, and some killer immunoglobulin (KIR) receptors [27, 49, 71, 72, 73].

Furthermore, CRC lines express different arrays of NK receptor ligands. Regarding the most resistant colon cancer lines, some evidence has demonstrated that Colo 205 cells are generally resistant to killing by natural killer cells due to high expression of HLA‐A, HLA‐B, and HLA‐C molecules (ligands of KIR receptors, [74]), CD155 (ligand for CD96 and TIGIT, inhibitory receptors; [75]), and low expression of MICA (ligand for NKG2D, one of the main activating NK receptors; [76]). HT‐29 cells express MICA on their membranes, but they are also characterized by a high expression of HLA‐ABC molecules, as well as HLA‐E, ligand of the inhibitory receptor NKG2A [77, 78, 79]. On the other hand, many studies have shown that RKO cells are sensitive to NK killing due to the expression of the NKG2D ligands MICB, ULBP1, ULBP2, and ULBP3, as well as the lack of HLA‐E expression [80, 81, 82, 83]. Caco‐2 line shows low expression of the MHC‐I complex and HLA‐E [79, 83], while no data are available on the expression of NK ligands on DiFi cells.

Another explanation for the differential sensitivity to iNKT cell killing might be given by the aneuploidy status of colon cancer cells. It has been observed that chromosome mis‐segregation causes the insurgence of complex karyotypes, and cells with these karyotypes are more efficiently killed by natural killer cells [84]. In line with these findings, we observed that two out of three of the most sensitive CRC lines (DiFi and Caco‐2) have the highest ploidy of the whole panel (Table S1). In the case of the RKO line, it must be considered that these cells are microsatellite instable, and it has been widely recognized that microsatellite‐instable tumors are usually more immunocompetent than the microsatellite‐stable counterparts [85, 86, 87]. Overall, this heterogeneity might also reflect the heterogeneity commonly found among patients, some responding better than others to immunotherapies [88].

In addition, we demonstrated that iNKT cells can eliminate patient‐derived CRC cells. This finding raises the question of how their cytotoxic activity is impaired during colon cancer progression. We speculate that this occurs as a consequence of changes in tissue architecture that might impede iNKT cells to form the immunological synapse with target cells, or the involvement of other components of the tumor microenvironment (tumor‐associated macrophages, neutrophils, gut microbiota, metabolites, etc.) in the modulation of iNKT cell activity toward an exhausted or protumorigenic phenotype. Nonetheless, the actual contribution of these factors will need further studies.

Altogether, these results might become of interest in the light of potential iNKT cell‐based immunotherapies against colon cancer. It has been observed that, even if iNKT cells are found in considerably low numbers [50], there is a higher infiltration in human CRC tumors [47]. Therapeutic strategies could be targeted to potentiate the killing activities of iNKT cells already infiltrating the tumor, or selecting iNKT cells with high cytotoxic potential for cell transfer interventions. Regarding the latter, as circulating and colon‐derived iNKT cells show similar killing efficiency, it would be more advantageous to obtain cells from peripheral blood, as numbers would be higher for further expansion, and obtaining surgical specimens might logistically become more difficult.

In conclusion, this study sheds light on the effector roles of human circulating and intestinal iNKT cells against colorectal cancer. Furthermore, it provides new knowledge about the activation and mechanistic cues of iNKT cell cytotoxicity, elucidating the importance of studying iNKT cell behavior to propose them as a new immunotherapeutic approach against colon cancer.

## 5 Conclusions

In this work, we demonstrated that both intestinal and circulating iNKT cells kill colon cancer cells in a similar manner as circulating NK cells. Furthermore, we showed that iNKT cells increase the release of soluble cytotoxic molecules upon encounter with target cells, being functional perforin and granzymes essential for iNKT cell cytotoxicity. In addition, we observed that antigen presentation by CRC cells is not required for their recognition by iNKT cell lines. To sum up, we explored the cellular requirements of human iNKT cell cytotoxicity in the context of colorectal cancer and concluded that iNKT cells might have a role in immunosurveillance in the colon via the recognition and killing of tumor cells.

## Conflict of interest

The authors declare no conflict of interest.

### Peer Review

The peer review history for this article is available at https://publons.com/publon/10.1002/1878‐0261.13104.

## Author contributions

ADB curated the data, analyzed, investigated, and wrote–original draft; CB investigated and conceptualized the study; GL curated the data and investigated the study; LB, FB, and AC provided resources; FC provided resources and wrote–review and editing; FF conceptualized and supervised the study, involved in funding acquisition and project administration, and wrote–original draft, review, and editing.

## Supporting information


**Table** **S1**. Cell lines used in the study.
**Table** **S2**. List of iNKT cell lines generated for functional studies.
**Table** **S3**. List of antibodies used for flow cytometry analysis.
**Table** **S4**. Antibodies used for ELISA assays.
**Fig. S1.** Flow cytometry analyses conducted in this study.
**Fig. S2.** iNKT cell lines are cytotoxic against colon cancer cell lines.
**Fig. S3.** Cytotoxicity effectors on NK cells.
**Fig. S4.** Effect of cytotoxicity mechanism and CD1d inhibition on cytotoxic mediators.
**Fig. S5.** iNKT cell killing of patient‐derived CRC cells.

## Data Availability

Data sharing is not applicable to this article as no new data were created or analyzed in this study.
