# Supplementary material for: Human intestinal and circulating invariant natural killer T cells are cytotoxic against colorectal cancer cells via the perforin–granzyme pathway
Source: Mol Oncol. 2021 Oct 21;15(12):3385–403. doi: 10.1002/1878-0261.13104 (PMC8637555; doi:10.1002/1878-0261.13104)
Supplement: Supplementary file 1 — Table S1. Cell lines used in the study. Table S2. List of iNKT cell lines generated for functional studies. Table S3. List of antibodies used for flow cytometry analysis. Table S4. Antibodies used for ELISA assays. Fig. S1. Flow cytometry analyses conducted in this study. Fig. S2. iNKT cell lines are cytotoxic against colon cancer cell lines. Fig. S3. Cytotoxicity effectors on NK cells. Fig. S4. Effect of cytotoxicity mechanism and CD1d inhibition on cytotoxic mediators. Fig. S5. iNKT cell killing of patient‐derived CRC cells. [file MOL2-15-3385-s001.pdf]

Supplementary Tables

Table S1. Cell lines used in the study. MSS: microsatellite-stable, MSI: microsatellite-instable, FAP: familial adenomatous polyposis

Cell Line	Origin	Properties	Medium
Caco-2	Colon, adenocarcinoma	Adherent, tetraploid, MSS	Minimum Essential Medium Eagle + 20% fetal bovine serum (FBS), 2 mM Glu, 1 mM NaP, 0.1 mM non-essential amino acids (NEAA)
Colo 205	Colon cancer metastatic site: ascites	Suspension, hyper-triploid, MSS	RPMI-1640 with 2 mM Glu, 10% FBS
DiFi	Colon, carcinoma from a FAP patient	Adherent, tetraploid, MSS	F-12 1X nutrient mix + 10% FBS
HT-29	Colon, adenocarcinoma	Adherent, triploid	Dulbecco's modified Eagle medium (DMEM) + 2 mM Glu, 10% FBS
RKO	Colon, carcinoma	Adherent, diploid, MSI	MEM + 20% FBS, 2 mM Glu, 1 mM NaP, 0.1 mM NEAA
NCM-460D	Normal colon	Adherent, diploid	F-12 1X nutrient mix + 10% FBS
NK-92	Natural killer, non-Hodgkin lymphoma	Suspension	RPMI-1640 with 2 mM Glu, 5% human serum, 100 IU/ml IL-2

Table S2. List of iNKT cell lines generated for functional studies.

Name	Organ	Individual Type
CD1	Colon	Crohn’s disease patient
CD2	Colon	Crohn’s disease patient
CD3	Colon	Crohn’s disease patient
NUN	Colon	Ulcerative colitis patient
PB1	Peripheral blood	Healthy donor
PB2	Peripheral blood	Healthy donor
PB3	Peripheral blood	Healthy donor
PB5	Peripheral blood	Healthy donor
PB6	Peripheral blood	Healthy donor

Table S3. List of antibodies used for flow cytometry analysis.

Antibody	Clone	Fluorochrome	Vendor
Anti-human CD1d:PBS-57 Tetramer	-	PE	NIH Tetramer Facility
Anti-human CD1d	51.1	APC	Biolegend
Anti-human CD45	HI30	BV510	BD Biosciences
Anti-human CD56	MY31	BV605	TONBO Biosciences
Anti-human CD95 (Fas)	DX2	BV650	Biolegend
Anti-human CD107A (LAMP-1)	H4A3	APC-Cy7	Biolegend

Anti-human CD178 (Fas Ligand)	NOK-1	APC	TONBO Biosciences
Anti-human EPCAM	1B7	PERCP	eBioscience
Anti-human Granzyme B	N4TL33	eF450	Invitrogen
Anti-human Perforin	dG9	BV510	Biolegend
Anti-human TRAIL	RIK-2	BV786	BD Biosciences
Anti-human TRAILR1	S35-934	BV605	BD Biosciences
Anti-human TRAILR2	B-K29	BV510	BD Biosciences

Table S4. Antibodies used for ELISA assays.

**Antibody**	**Clone**	**Vendor**
Anti-human granzyme B, purified	GB10	Mabtech
Anti-human granzyme B, biotinylated	GB11	Mabtech
Anti-human perforin, purified	Pf-80/164	Mabtech
Ani-human perforin, biotinylated	Pf-344	Mabtech

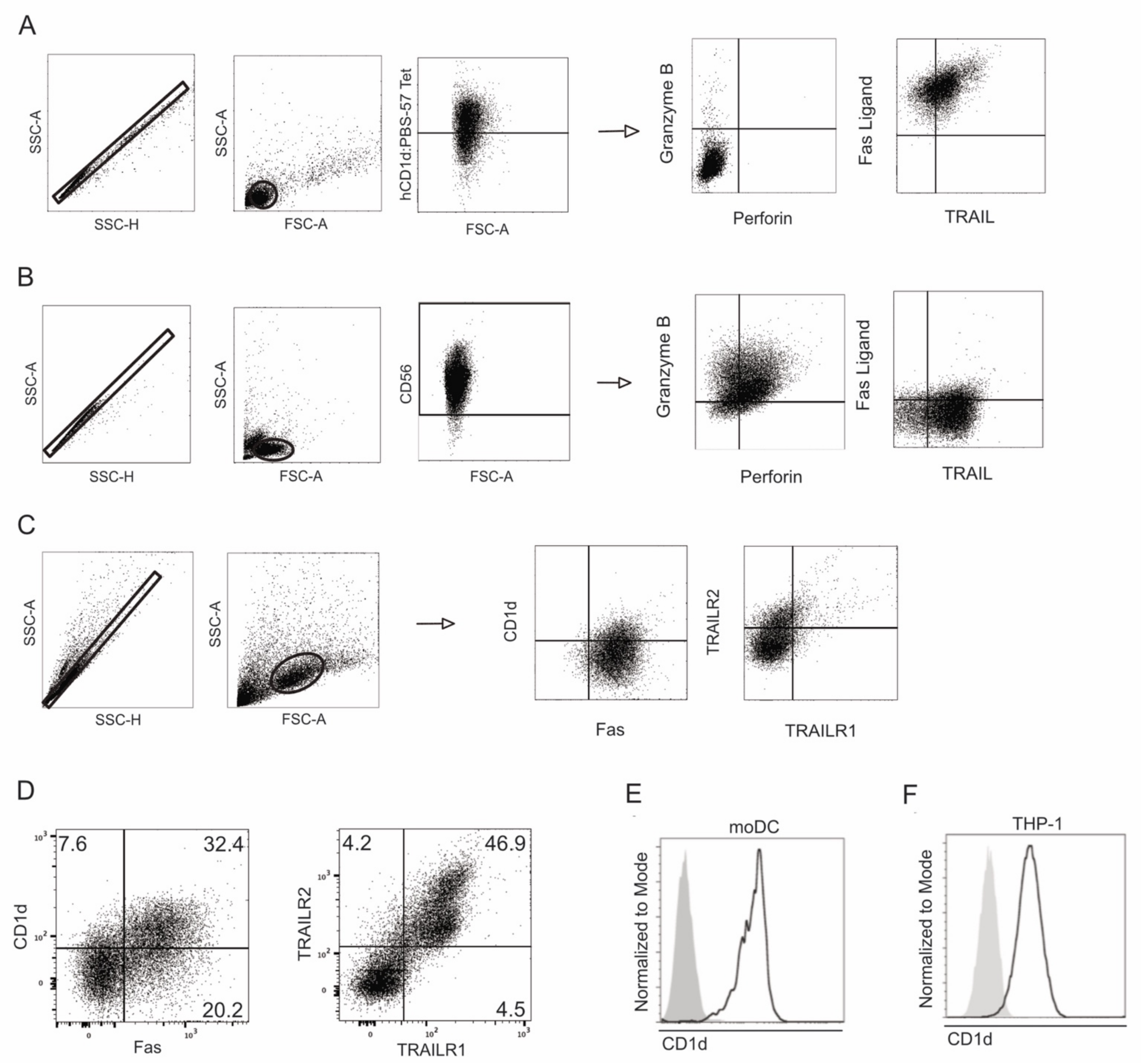

Figure S1. *Flow cytometry analyses conducted in this study*. Gating strategies for A. iNKT cells, B. NK cells, and C. CRC cells. D. Representative dot plots for CRC cell analyses. E-F. CD1d expression by dendritic cells (E) and THP-1 cells (F).

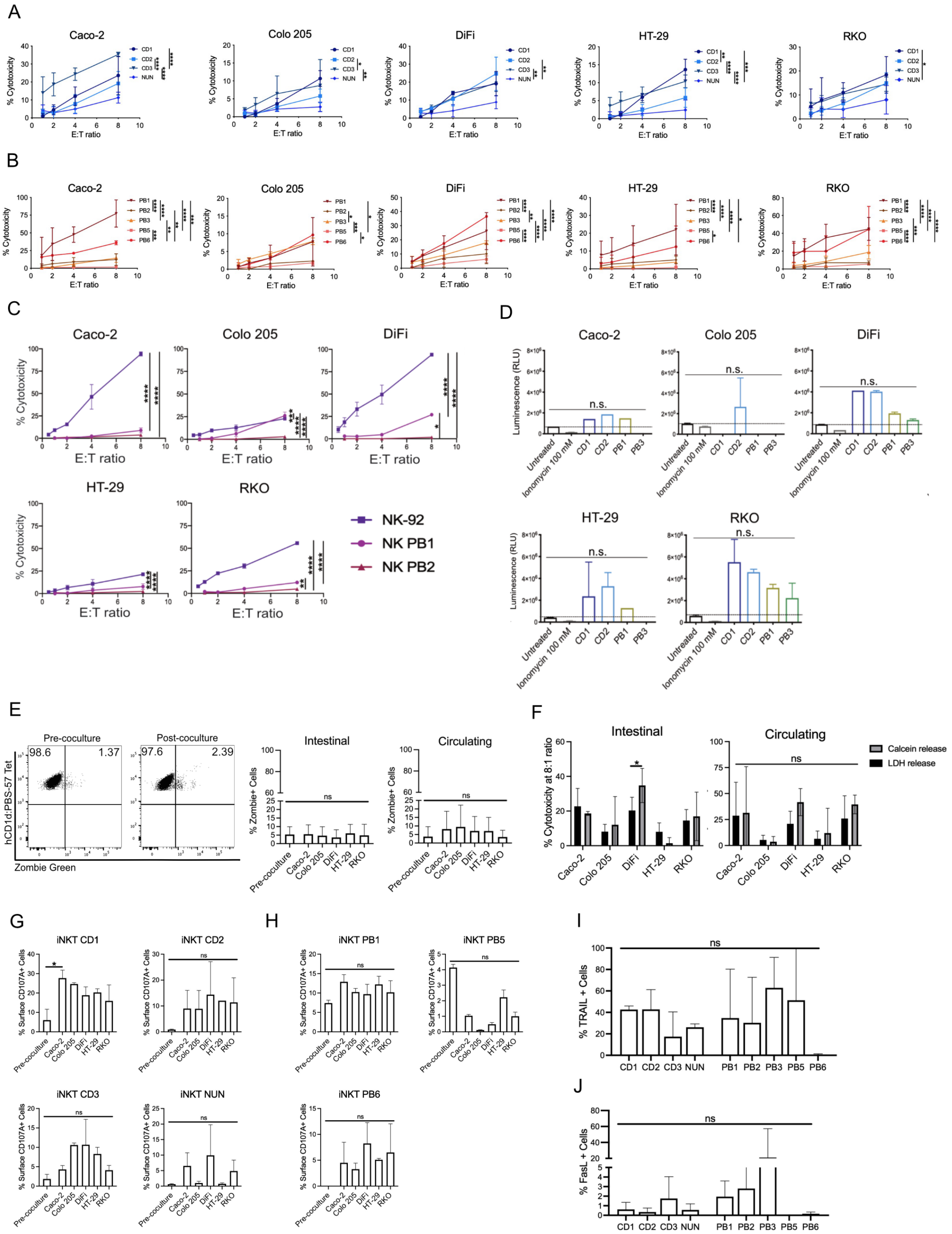
A
Caco-2
Colo 205
DiFi
HT-29
RKO
% Cytotoxicity
E:T ratio
CD1
CD2
CD3
NUN
B
PB1
PB2
PB3
PB5
PB6
C
NK-92
NK PB1
NK PB2
D
Luminescence (RLU)
n.s.
Untreated
Ionomycin 100 mM
E
Pre-coculture
Post-coculture
98.6
1.37
97.6
2.39
hCD1d:PBS-57 Tet
Zombie Green
Intestinal
Circulating
% Zombie+ Cells
ns
F
% Cytotoxicity at 8:1 ratio
Calcein release
LDH release
G
iNKT CD1
iNKT CD2
iNKT CD3
iNKT NUN
% Surface CD107A+ Cells
H
iNKT PB1
iNKT PB5
iNKT PB6
I
% TRAIL + Cells
J
% FasL + Cells

Figure S2. *iNKT cell lines are cytotoxic against colon cancer cell lines*. A-D. Killing activities by effector cells per target cell line. A. Intestinal iNKT cells. B. Circulating iNKT cells. D. NK cells. D. Caspase 3/7 activity by colon cancer cells upon coculture with iNKT cells. E. Cell viability of iNKT cells pre- and post-coculture with CRC cell lines. F. Graphs comparing cytotoxicity by intestinal (left) and circulating (right) iNKT cells measured by LDH release vs calcein release at 8:1 E:T ratio. G-H. Surface CD107A frequencies for colon (G) and peripheral blood (H) iNKT cell lines. I-J. TRAIL (I) and Fas Ligand (J) frequencies on iNKT cells at steady-state conditions. Two-way ANOVA test was used to assess statistical significance and Tukey test for multiple comparisons in A, B and C; Kruskal-Wallis test was used for statistical significance and Dunn's test for multiple comparisons in D-J. Data are means ± SD of at least 3 independent experiments except for blood NK cells. n.s. non-significant, p-value < 0.05 (*), 0.01 (**), 0.001 (***), 0.0001 (****).

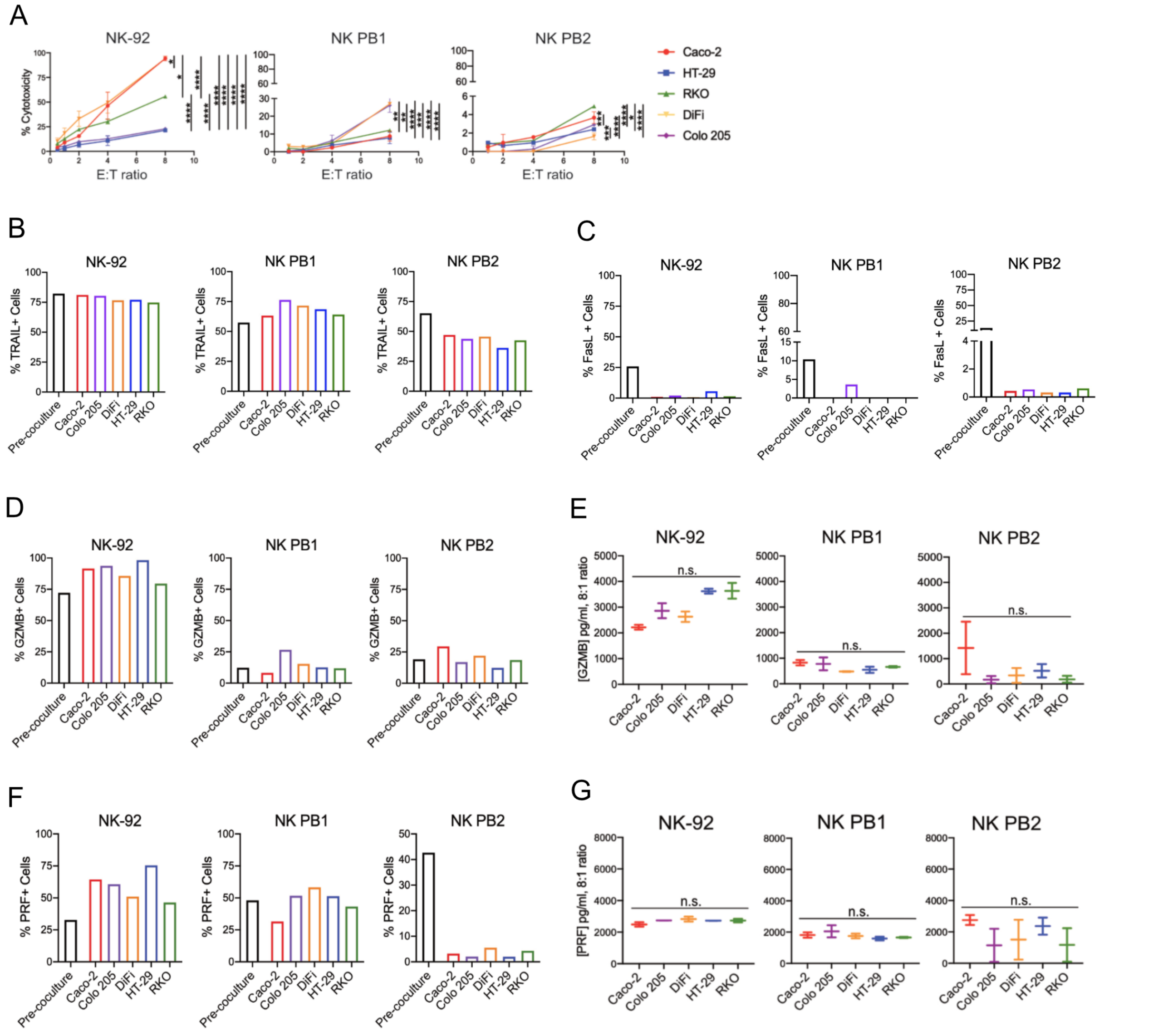

Figure S3. *Cytotoxicity effectors on NK cells*. A. E:T ratio curves. B. TRAIL, C. Fas Ligand positive cells. D-E. Granzyme B frequencies (D) and release in supernatants (E). F-G. Perforin frequencies (F) and release in supernatants (G). Representative data are presented for B-G.

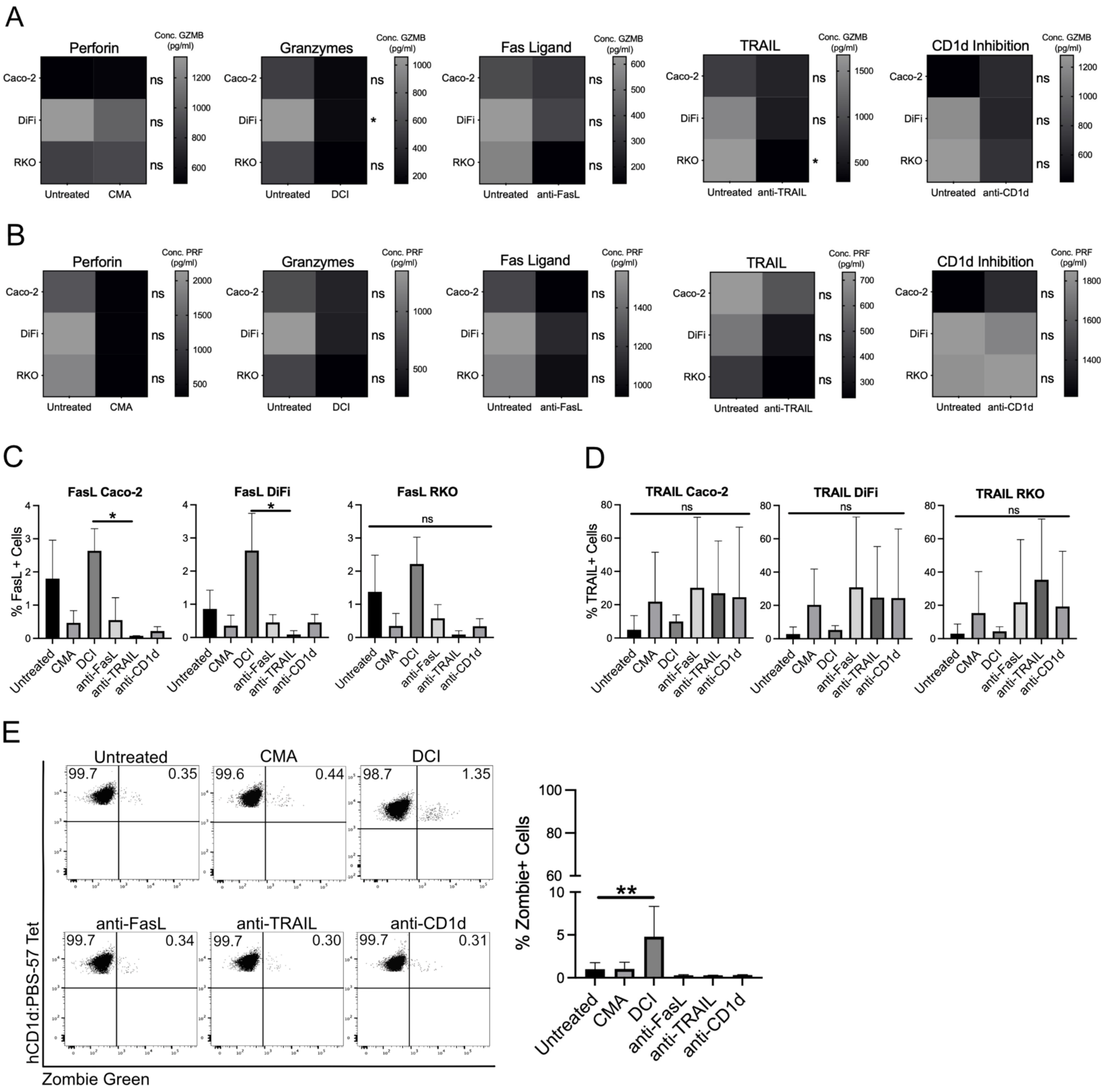

Figure S4. *Effect of cytotoxicity mechanism and CD1d inhibition on cytotoxic mediators.* A. Granzyme B concentration; B. Perforin concentration; C. Fas ligand expression; D. TRAIL expression. E. Cell viability. Data are means in A and B and means ± SD in C-E of 3 independent experiments from five iNKT cell lines. Two-way ANOVA tests were used to assess statistical significance and Šidak's tests for multiple comparisons on A and B, whereas Kruskal-Wallis test was used for statistical significance and Dunn's test for multiple comparisons in C-E. ns. non-significant, p-value < 0.05 (*), 0.01 (**), 0.001 (***), 0.0001 (****).

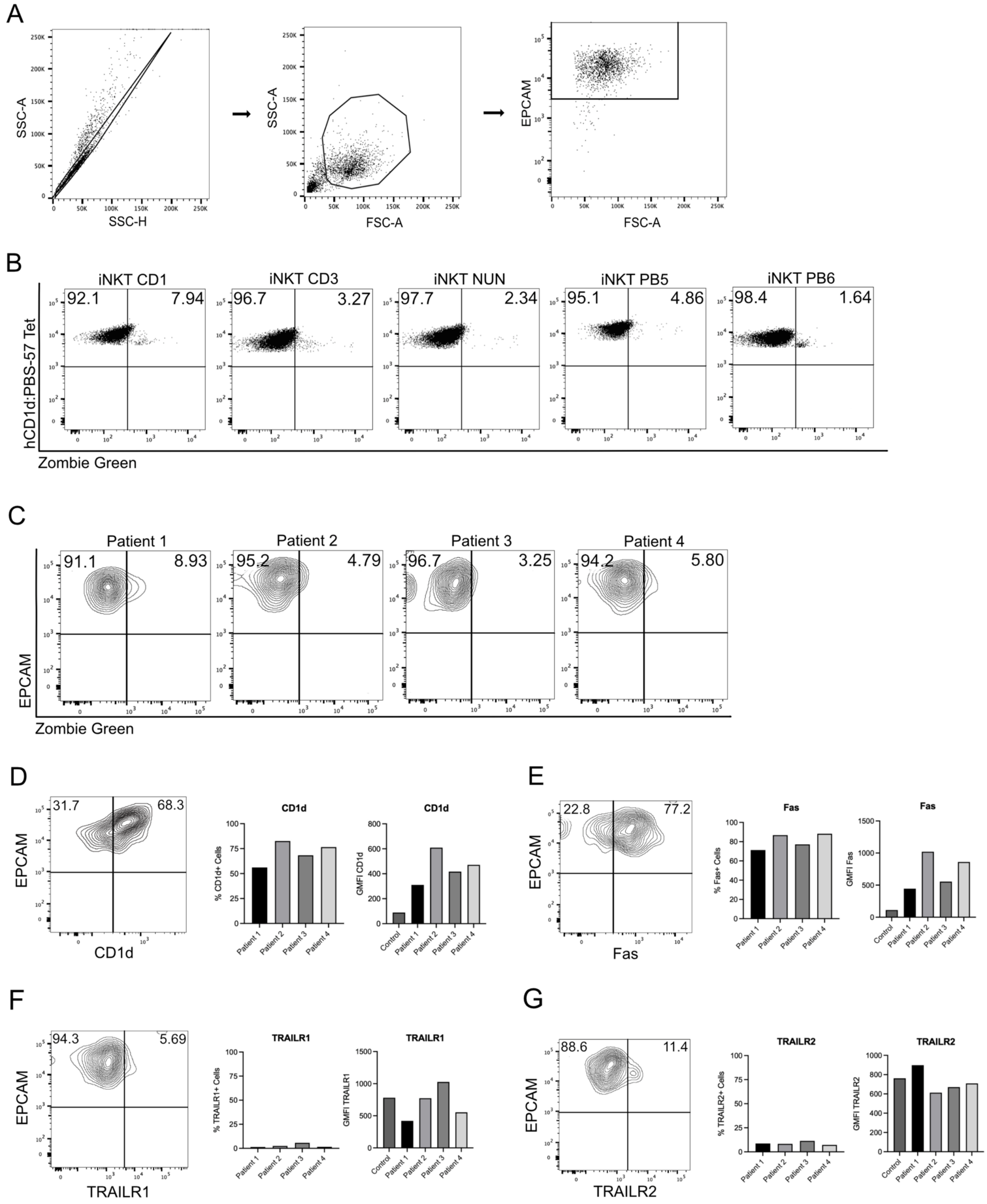

Figure S5. *iNKT cell killing of patient-derived CRC cells*. A. Gating strategy for the analysis of patient CRC cells. B. Cell viability of the iNKT cell lines used for this set of experiments prior to coculture. C. Cell viability of fresh, patient-derived CRC cells used prior to coculture. D-G. Expression of CD1d (D), Fas (E), TRAILR1 (F), and TRAILR2 (G) by CRC cells.
